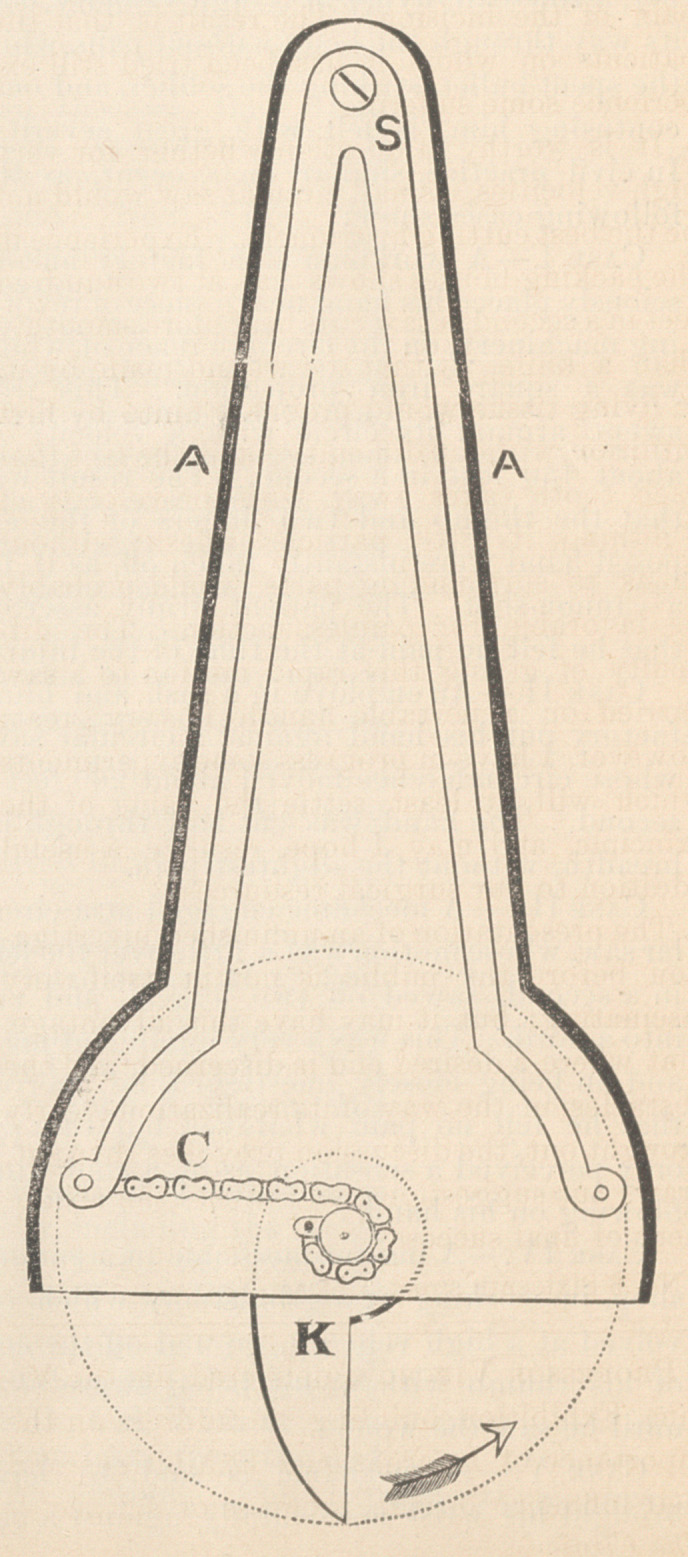# Studies and Experiments on Producing Painless Incisions without Anæsthetics

**Published:** 1873-11-15

**Authors:** Edmund Andrews

**Affiliations:** Professor of Surgery in Chicago Medical College; No. 6 Sixteenth street, Chicago


### Original Communications.

### STUDIES AND EXPERIMENTS ON PRODUCING PAINLESS INCISIONS WITHOUT ANÆSTHETICS.

BY EDMUND ANDREWS, M.D., PROFESSOR OF SURGERY IN CHICAGO MEDICAL COLLEGE.

Mechanical injuries received from bodies moving at a high velocity are painless. The cannon-ball which, under full motion, takes away a limb, occasions but little sensation, while one which strikes with a slow motion may cause acute suffering. In the same manner, a rifle-ball, flying at a high velocity, cuts its way through the body without pain, while the spent bullet, striking the soldier, and only contusing him, is felt with great severity. In civil practice, similar facts occur, as the following cases show:

Case I.—A workman in a factory unconsciously placed his hand near a piece of revolving machinery, on the circumference of which was a square iron projection. This iron swept around its circle with a velocity of about 150 feet in a second. The result was that the thumb and two fingers of the exposed hand were instantly taken off, as if by a cannon-shot. The patient firmly asserted that he felt no pain at the time of the injury.

Case II.—An employe in a sash and blind factory put his hand against a circular saw, whose circumference moved about 200 feet a second. The hand was cut half through its breadth, without the slightest pain.

Case III.—A mechanic engaged at a circular saw, whose motion was a little over 100 feet in a second, sawed off two fingers, and cut into a third. This was a very intelligent man, and on being closely questioned, he averred that he felt no pain whatever, but that he only perceived a sensation as of a cool wind blowing on his hand.

Case IV.—A master-mechanic in a cutlery shop, in working at a grindstone, which revolved at a high velocity, ground off the end of his thumb without his being aware of it until he saw the wound.

Case V.—A mechanic is said to have brought his wrist accidentally against a circular-saw, cutting it nearly off, without the slightest pain. Finding that the member was nearly severed, rendering its restoration hopeless, and observing that the injury occasioned no suffering, he deliberately placed the uncut portion against the saw, and thus voluntarily completed the amputation.

It is the universal testimony of mechanics, that incisions made by saws moving from 100 to 200 feet per second are absolutely painless. Thus, in one of the great packing-houses of Chicago, the carcasses of the slaughtered animals are cut up for packing, by a circular saw revolving at about 200 feet per second. At this velocity the saw cuts the meat *more smoothly than a knife*, and the workmen, who are occasionally wounded by it, uniformly assert that the incisions are wholly painless.

From various observations, I judge that the minimum velocity necessary to make a saw-cut without pain is between 100 and 150 feet per second. Probably the velocity of a bullet wounding painlessly must be somewhat greater, as it is a blunter object. The ordinary army musket-ball has a velocity, when first leaving the gun, of 1700 feet in a second; but the motion is rapidly checked by the resistance of the air, so that the minimum velocity of a bullet wounding painlessly may be only 600 or 700 feet per second, or even less. These facts render it evident that the human flesh may be cut or torn without pain by any object moving between 150 and 1000 feet in a second.

Several surgical instruments have been in use, designed to diminish pain by increased velocity. The spring-vaccinator, the spring-lancet, and the various kinds of scarificators are examples of this class; but none of them make any pretense of obtaining complete painlessness, unless applied on surfaces where from dropsy, or other causes, they act on tissues possessing diminished sensibility. From my tests, I do not think any of them attain to one-third the swiftness requisite for painless incisions. Reflection upon these facts has induced me to commence a series of experiments to determine whether it is possible to devise an instrument small enough for convenience in surgery, and swift enough to cut without pain. These experiments have not yet been fully successful; but their results give hope that further research will enable us yet to introduce into surgery the important improvement of *anæsthesia by velocity*. Two obstacles have confronted me from the outset:

1. The great difficulty of obtaining a motion of 150 feet per second in a small instrument.

2. The increased sensitiveness of a surgical patient from the inflammation of the parts to be operated on, and from the concentration of his attention on the expected incisions. Both of these circumstances are absent from accidental, painless saw-cuts and bullet-wounds. This hyper-sensitiveness demands a sufficient increase of velocity to overcome it. My first experiment was made by means of a sharp instrument for opening abscesses, which, by a rubber spring, was made to shoot out a blade endwise, like Johnson's scarificator for dropsical limbs. I used a spring of perhaps fifteen pounds tension; and the testimony of the patients was, that the pain of the incision was slight, but still perceptible. Observation upon the violent jerk which the knife received at its stopping-point, in consequence of the power of this spring, satisfied me that this instrument was not safe, for sooner or later the repetition of shocks would develop cracks in the steel, and finally cause the blade to break loose and shoot like an arrow into the body, possibly wounding some vital part. I therefore had one constructed with a rubber spring of twenty pounds tension, which coiled around a small shaft, on whose extremity was set, at right angles, a thin knife. A key wound it up, and a trigger set it free, by which means the knife swept a circle with great velocity, without any risk of shooting endwise like an arrow in case of breakage. To measure the velocity, I caused a wheel to be made two feet in diameter, which revolved with a known velocity. From the circumference of this wheel projected two continuous rims of paper, parallel to each other, and one inch apart. The wheel being set in motion, and its velocity ascertained, the knife was made to cut across the edges of the paper, notching the tw rims. Of course, the time occupied by the knife in passing from one rim to the other was shown by the fact that the notches were not opposite, but the second rim was cut further back than the first, and the distance served as a datum for calculating the velocity. To my disappointment, I found I had only attained a motion of twenty-nine feet in a second, which was only one-fifth of what I desired. The result was still a remnant of pain, when applied to an abscess or a hydrocele. I next ordered an instrument made with a steel spring having fifty pounds tension, which is here represented.

S is the spring; K is the knife, attached to a shaft which is made to revolve by the traction of the chain C. The motion is in the direction of the arrow. A A is a case which encloses the whole machinery, but which is represented as absent from the front, in order to show the internal structure. The knife is liberated by a trigger, and the depth of the incision is regulated by a sliding foot to the instrument. The sweep of the knife is so rapid as to be almost invisible, and it cuts a hair in mid-air without difficulty, yet it moves only about seventy feet in a second, which is less than half what is necessary to abolish the pain of the incision. The result is, that the patients on whom it has been tried still experience some suffering.

It is worthy of question whether, for very high velocities, a small circular saw would not be the best cutting instrument. Experience in the packing-houses shows that, at two hundred feet in a second, a saw cuts beef more smoothly than a knife, so that the wound made by it, in living tissue would probably unite by first intuition without difficulty; and the fact that each tooth takes away, with the velocity of lightning, its little particle of flesh; without shock to surrounding parts, would probably be favorable for painless action. The difficulty of giving this rapid motion to a saw carried on a portable handle is very great; however, I have in progress some experiments which will at least settle the value of the principle, and may, I hope, result in a useful addition to our surgical resources.

The presentation of an unfinished investigation before the public is not in itself very fascinating; but it may have this advantage, that where a desired end is discerned, and the obstacles in the way of its realization clearly brought out, the discussion provokes thought, draws out suggestions, and favors the attainment of final success.

No. 6 Sixteenth street, Chicago.

---

Professor Virchow delivered, in the Vienna Exhibition building, an address on the importance of International Exhibitions and their influence on the progress of science.—*The Clinic.*

## Figures and Tables

**Figure f1:**